# Awakening sleeping beauty: production of propionic acid in *Escherichia coli* through the *sbm* operon requires the activity of a methylmalonyl-CoA epimerase

**DOI:** 10.1186/s12934-017-0735-4

**Published:** 2017-07-17

**Authors:** Ricardo Axayacatl Gonzalez-Garcia, Tim McCubbin, Annalena Wille, Manuel Plan, Lars Keld Nielsen, Esteban Marcellin

**Affiliations:** 1 Australian Institute for Bioengineering and Nanotechnology, The University of Queensland, Brisbane, QLD 4072 Australia; 2 Bielefeld University, Universitätsstraße 25, 33615 Bielefeld, Germany

**Keywords:** Propionic acid, Methylmalonyl-COA epimerase, *Propionibacterium acidipropionici*, *Escherichia coli*

## Abstract

**Background:**

Propionic acid is used primarily as a food preservative with smaller applications as a chemical building block for the production of many products including fabrics, cosmetics, drugs, and plastics. Biological production using propionibacteria would be competitive against chemical production through hydrocarboxylation of ethylene if native producers could be engineered to reach near-theoretical yield and good productivity. Unfortunately, engineering propionibacteria has proven very challenging. It has been suggested that activation of the sleeping beauty operon in *Escherichia coli* is sufficient to achieve propionic acid production. Optimising *E. coli* production should be much easier than engineering propionibacteria if tolerance issues can be addressed.

**Results:**

Propionic acid is produced in *E. coli* via the sleeping beauty mutase operon under anaerobic conditions in rich medium via amino acid degradation. We observed that the *sbm* operon enhances amino acids degradation to propionic acid and allows *E. coli* to degrade isoleucine. However, we show here that the operon lacks an epimerase reaction that enables propionic acid production in minimal medium containing glucose as the sole carbon source. Production from glucose can be restored by engineering the system with a methylmalonyl-CoA epimerase from *Propionibacterium acidipropionici* (0.23 ± 0.02 mM). 1-Propanol production was also detected from the promiscuous activity of the native alcohol dehydrogenase (AdhE). We also show that aerobic conditions are favourable for propionic acid production. Finally, we increase titre 65 times using a combination of promoter engineering and process optimisation.

**Conclusions:**

The native *sbm* operon encodes an incomplete pathway. Production of propionic acid from glucose as sole carbon source is possible when the pathway is complemented with a methylmalonyl-CoA epimerase. Although propionic acid via the restored succinate dissimilation pathway is considered a fermentative process, the engineered pathway was shown to be functional under anaerobic and aerobic conditions.

**Electronic supplementary material:**

The online version of this article (doi:10.1186/s12934-017-0735-4) contains supplementary material, which is available to authorized users.

## Background

In 2012, the US Department of Energy identified propionic acid (PA) as one of the top 30 building blocks to be produced biologically due to its versatility, significance, and the potential for large-scale biological production [1]. PA is a colourless, oily-compound used as a preservative in cereals and animal feeds. Recently, PA applications have expanded to include uses in the textile, dye, drugs, cosmetic and plastic industries [2]. In 2014, the global market for PA was estimated to be USD 1.07 billion with a total volume of 400,000 tonnes but is expected to reach USD 1.55 billion by 2020 [2]. PA is currently produced from petrochemical processes. However, consumers are asking for sustainable alternatives. Biological production of PA addresses many sustainability concerns including reduced energy demand as well as being sourced biologically, a desirable attribute in the food industry [3].

PA can be produced biologically through two main native metabolic pathways, the Wood-Werkman cycle of propionibacteria [4, 5] and the acrylate cycle of clostridia [6]. Native PA producers, such as propionibacteria, can produce PA as their primary fermentation product achieving high yields. However, these organisms exhibit slow growth, require fastidious nutrient media for growth and lack reliable tools for metabolic engineering. As a result, rational design of native PA producers remains challenging. Only a few examples exist in the literature and these typically result in modest improvements and occur in lower producing native producers that are easier to engineer, such as *P. shermanii* [7, 8]. Furthermore, these improved strains are still inferior to the best native producers. Heterologous production of PA represents an attractive alternative to overcome limitations in metabolic engineering of native producers and offers a valuable tool to test rational hypotheses that cannot be tested in native producers due to our inability to engineer them [6].

**Fig. 1:**
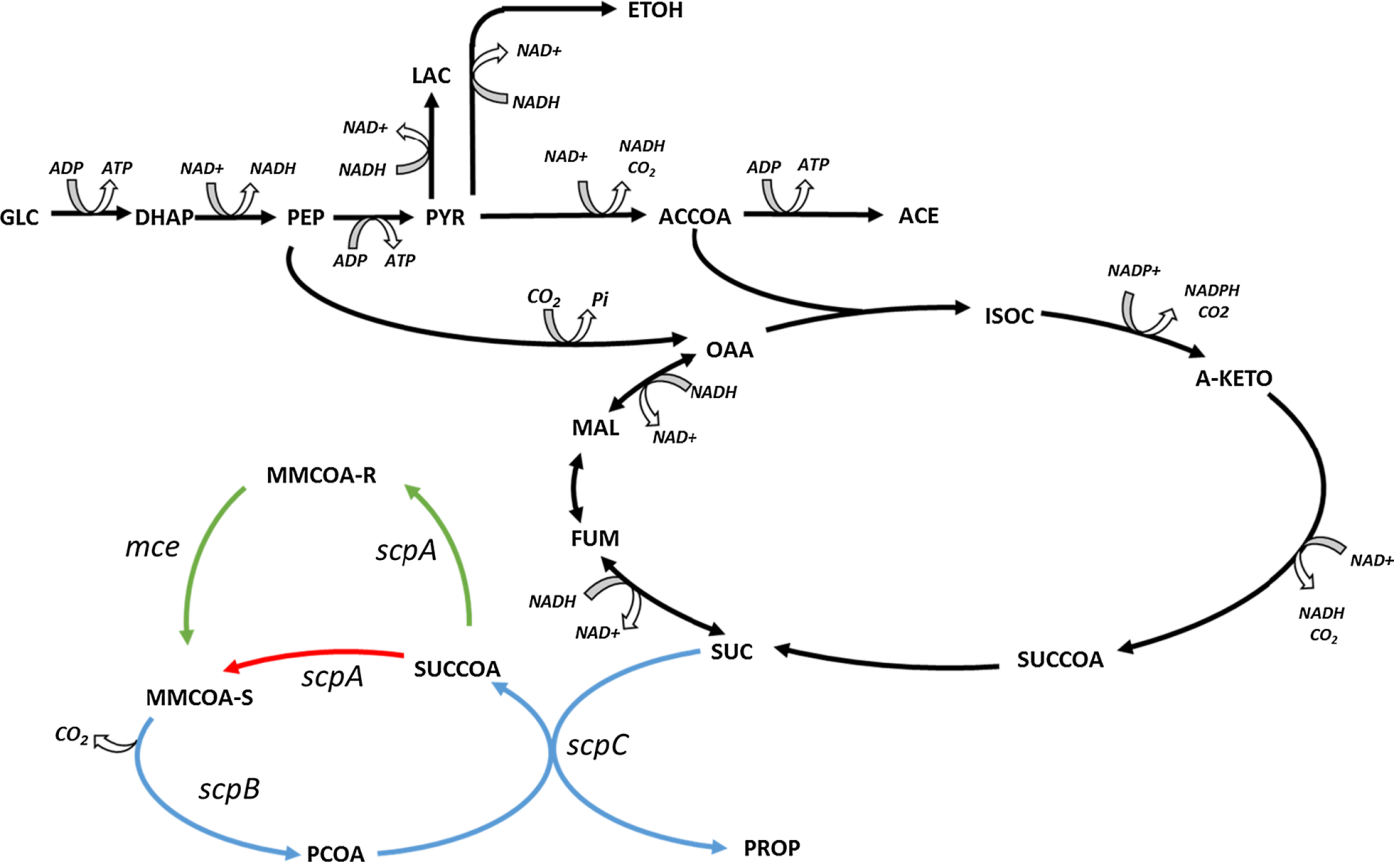
Succinate dissimilation pathway for PA production. Principal reactions in *E. coli’s* central carbon metabolism are shown in *black*. The reported succinate dissimilation pathway encoded in the *sbm* operon is shown in *blue*. The reactions to complement the succinate pathway presented in this work appear in *green*. *GLC* glucose, *DHAP* dihydroxyacetone phosphate, *PEP* phosphoenolpyruvate, *PYR* pyruvate, *ETOH* ethanol, *ACCOA* acetyl-CoA, *ACE* acetate, *OAA* oxaloacetate, *ISOC* isocitrate, *A_KETO* α-ketoglutarate, *SUCCOA* succinyl-CoA, *SUC* succinate, *FUM* fumarate, *MAL* malate, *MMCOA-S/R* (R/S)-methylmalonyl CoA, *PCOA* propionyl-CoA, *PROP* propionate

*Escherichia coli* is a robust microorganism. The heterologous production of organic acids and alcohols by *E. coli* has proven to be a suitable option as evidenced by the commercial success of succinate production [9] by Requette, Bioamber, Reverdia and Myriad [10, 11]; 1,3-propanediol (PDO) by DuPont Tate & Lyle [12–14]; and 1,4-butanediol (BDO) by Genomatica [15–17]. Amongst the many products that have been produced in *E. coli,* PA was first produced in *E. coli* by Kandasamy et al. [6] using the acrylate pathway. More recently, several investigations have reported PA production using the silent native operon known as the sleeping beauty mutase, *sbm* [18–22]. The *sbm* operon encodes four enzymes, three of them allegedly able to complete a succinate dissimilation cycle: methylmalonyl-CoA mutase (ScpA), biotin-independent methylmalonyl-CoA carboxylase (ScpB), and propionyl-CoA:succinate CoA transferase (ScpC) (Fig. 1) [23]. The fourth gene in the operon (*argK*) corresponds to a membrane-bound ATP kinase, and its role in the cycle has not yet been elucidated.

PA production in *E. coli* expressing the operon [18–22] indicates that ScpA, ScpB and ScpC are sufficient to form the cycle (Fig. 1, red). Indeed, in studying the kinetics of the enzymes in vitro, Haller et al. coupled ScpA (formerly Sbm) to ScpB and observed the two-step conversion of succinyl-CoA into propionyl-CoA [23]. However, this step is not obvious given that methylmalonyl-CoA decarboxylases are reported to act on the (S)-form of methylmalonyl-CoA [23], whereas the mutase in *E. coli* has been shown to produce the (R)-form [24]. In agreement with Kannan et al. [24], Dayem et al. [25] confirmed that ScpA catalyses the stereospecific conversion of (R)-methylmalonyl-CoA into succinyl-CoA. As such, an additional enzymatic step—an epimerase—would be required to complete the cycle from succinyl-CoA into PA to make the silent operon functional (Fig. 1). Hence, we set out to determine whether the additional epimerase step was required.

We used an in silico metabolic reconstruction to evaluate the feasibility of using the reported succinate dissimilation cycle encoded in the *sbm* operon for PA production. *In silico* results show that PA can be produced only when the operon is active and the methylmalonyl-CoA decarboxylase is assumed to be active on the (R)-methylmalonyl-CoA enantiomer. However, our experimental results indicate that for PA to be produced, an additional epimerase is required. Introduction of the methylmalonyl-CoA epimerase from *Propionibacterium acidipropionici* enabled PA production, from sugar as the sole carbon source, under anaerobic and aerobic conditions.

## Methods

### *In silico* analysis

The genome-scale model *iJO1366* for *E. coli* K12 MG1655 [26] was used for in silico simulations. Flux balance analysis was performed using the COBRA Toolbox 2.0.5 [27] and MATLAB R2014a. Reactions were deleted from the model by setting the reaction upper and lower bounds to zero. The lower maintenance bound was set to zero for non-growth related simulations. Finally, a PA transporter was added to allow the exchange of PA between the cytoplasmic and periplasmic compartments. All simulations were performed on minimal media with glucose as a sole carbon source assuming anaerobic conditions.

### Strains and plasmids

The full list of strains and plasmids can be found in Table 1. *E. coli* strains BL21 (DE3), and K12-MG1655seq+ were obtained from ATCC. *E. coli* strain DH5-alpha (BIOLINE) was used for regular cloning and fermentations.

**Table 1:** Bacterial strains and plasmids used

Strain/Plasmid	Description	Reference
*Escherichia coli* BL21 (DE3)	Expression host	Bioline
*Escherichia coli* DH5α	Cloning and expression host	Bioline
*Escherichia coli* MG1655	DNA template	CGSC^a^ 7740
pET28a+	Cloning vector	LifeTechnologies
pBR322	Cloning vector	LifeTechnologies
pMA_*mce*	pMA derived plasmid. Amp^R^ P_T7_-P_nar_-*mce*-t_T7_	LifeTechnologies
pET28_*sbm*	pET28a+ derived plasmid. Kan^R^-P_T7_-sbm-t_T7_	This work
pET28_*sbm*-*mce*	pET28a+ derived plasmid. Kan^R^-P_T7_-sbm-mce-t_T7_	This work
pBRP_BAD_	pBR322 derived plasmid Amp^R^. araC-P_BAD_	This work
pBRP_BAD__*sbm*	pBR322 derived plasmid Amp^R^. araC-P_BAD_-sbm	This work
pBRP_BAD__*sbm*-*mce*	pBR322 derived plasmid Amp^R^. araC-P_BAD_-sbm-mce	This work

^a^ Purchased from the Coli Genetic Stock Centre (CGSC)

### Construction of plasmids

*pET28_sbm* The 5.6 kb region containing the *sbm* operon was PCR amplified from *E. coli* K12-MG1566seq+ strain using primers sbmF1 and sbmR1 (Table 2). The PCR product was then digested with *Xba*I and *Hind*III restriction enzymes and cloned into the *Xba*I and *Hind*III sites in pET28a+. Confirmation primers listed in Table 2 were used to verify assembly.

**Table 2:** Primers

Primer name	Sequence	Description
sbmF1	CCTTAAGTCTAGAAGGAGAAAACCGATGTCTAACGTGCAGGAG	Forward primer for *sbm* region amplification
sbmR1	GCATGCCAAGCTTGCGGCCGCACTAGTTTATTAACCCAGCATCGAGCC	Reverse primer for *sbm* region amplification
P_BAD_F1	GATATCAATCGATTCTAGATTATGACAACTTGACGGC	Forward primer for *mce* gene amplification
P_BAD_R1	GATATCAAAGCTTGCGGCCGCTTATTACATCGGTTTTCTCCTACTAGTAGCTCGAATTCCCAAAAAAAC	Reverse primer for *mce* gene amplification
cP_BAD__F	TTCTCATGTTTGACAGCTTATCATCGATTC	Confirmation forward primer for PBAD region insertion in pBR322
cP_BAD__R	CACGGTGCCTGACTGCGTTAG	Confirmation reverse primer for PBAD region insertion in pBR322
cPET-sbmF1	CGAGCCCGATCTTCCCCATC	Confirmation forward primer for *sbm* region insertion in pET38a+
cPET-sbmR1	TGGTCCACGGTTGGGCGG	Confirmation reverse primer for *sbm* region insertion in pET38a+
cPET-sbmF2	GCGCCCGCACTATCATTG	Confirmation forward primer for sbm region insertion in pET38a+
cPET-sbmR2	ATCCGGATATAGTTCCTCCTTTCAGC	Confirmation reverse primer for sbm region insertion in pET38a+
c_sbm-mceF1	CCATGTTGATCACAGCGAAC	Confirmation forward primer for *mce* gene insertion in plasmid pET_sbm
C_sbm-mceR1	GTATTTCGGGTGCAGGAAGA	Confirmation reverse for *mce* gene insertion in plasmid pET_sbm

*pMA_mce* The methylmalonyl-CoA epimerase gene sequence (*PACID*_*19260*) [28] from *P. acidipropionici* was codon optimised using the “one codon-one amino acid” method in the online tool OPTIMISER [29]. The optimised sequence was renamed *mce* and introduced in a synthetic operon consisting of a T7 promoter, a strong ribosomal binding site (RBS), the coding sequence, an additional stop sequence TAA, and a T7 terminator. The designed operon was chemically synthesised (GeneArt, LifeTechnologies) and inserted in a pMA-derived plasmid.

*pET28_sbm-mce* The *mce* gene was PCR amplified from the plasmid pMA_mce using the primers mceF1 and mceR1. The PCR product was digested using *Xba*I and *Hind*III and cloned into the *Spe*I and *Hind*III sites in pET28_sbm-mce. Confirmation primers listed in Table 2 were used to verify assembly.

*pBRP*_*BAD*_ *_sbm-mce* The *araC*-P_*BAD*_ region was PCR amplified from the plasmid pKD46 with primers P_BAD_F1 and P_BAD_R1, digested with *Cla*I and *Hind*III and cloned into the *Cla*I and *Hind*III sites in pBR322 to build plasmid pBRP_*BAD*_. The *sbm*-*mce* fragment was released from pET28_sbm-mce by digestion with *Xba*I-*Hind*III and the fragment retrieved from a 1% agarose gel, purified and cloned at the *Spe*I-*Hind*III sites in the plasmid pBRP_*BAD*_. Confirmation primers listed in Table 2 were used to verify assembly.

PCR reactions were performed using Phusion DNA *Taq* polymerase (New England Biolabs). All PCR products and DNA fragments were purified using QIAGEN PCR Purification kit according to the manufacturer instructions. For the recovery of a DNA digestion product, DNA was loaded onto an agarose 1% gel, and the desired fragment was recovered using QIAGEN MinElute gel recovery kit following the manufacturer’s instructions. Ligations were performed using T4 DNA ligase at 16 °C for 16 h. Colony PCR was carried out to verify that the operon was in-frame using the primers listed in Table 2. The sequence was confirmed by Sanger sequencing (AGRF, Australia).

### Media composition and fermentations

Yeast extract and bacto-tryptone were obtained from BD Diagnostics Systems (Franklin Lakes, NJ). All other media components were purchased from Sigma-Aldrich Co. (St. Louis, MO). Three different culture media were used: complex LB medium (10 g/L yeast extract, 5 g/L bacto-tryptone, 5 g/L NaCl, pH 7.0; sterilized by autoclaving); complex SOC medium (20 g/L bacto-tryptone, 5 g/L yeast extract, 0.6 g/L NaCl, 0.5 g/L KCl, 0.952 g/L MgCl2, 1.204 MgSO4, 3.6 g/L glucose, pH 7.0; glucose and MgSO4 were filter sterilized and supplied to the medium after being autoclaved); and a modified M9 mineral medium [1.5 g/L KH_2_PO_4_, 4.34 g/L K_2_HPO_4_, 0.4 g/L (NH_4_)_2_SO_4_, 20 g/L glucose, 0.31 g/L MgSO4·7H2O, 1.5 mL/L vitamins solution, 1.5 mL/L trace elements solution, pH 7.0; medium was autoclaved; glucose and MgSO4·7H_2_O were autoclaved separately]. Trace elements solution (27 g/L FeCl_3_·6H_2_O, 2 g/L ZnCl_2_·4H_2_O, 2 g/L CaCl_2_·6H_2_O, 2 g/L Na_2_MO_4_·2H_2_O, 1.9 g/L CuSO_4_·5H_2_O, 0.5 g/L H_3_BO_3_, 100 mL HCl) and vitamins solution (0.42 g/L riboflavin, 5.4 g/L pantothenic acid, 6 g/L Niacin, 1.4 g/L pyridoxine, 0.06 g/L biotin, 0.04 g/L folic acid, 10 mg/L thiamine, 0.27 mg/L cobalamin) were filter sterilized and then supplemented to the media.

*Serum bottle fermentations* Recombinant *E. coli* strains were streaked on LB agar plates with antibiotics (Ampicillin 100 µg/mL) and incubated for 16 h at 37 °C. A single colony was picked and used to inoculate a 250 mL baffled flask with 50 mL of minimal media plus antibiotics. The overnight culture was used to inoculate a 160 mL serum bottle containing 100 mL of minimal media plus antibiotics. Arabinose (10 mM) and IPTG (1 mM) were added to the cultures where indicated. Serum bottles were flushed with N_2_ for 10 min before inoculation and incubated in an orbital shaker at 30 °C and 200 rpm. Samples were taken regularly. Optical density (OD) was measured at 600 nm (OD_600_) before samples were filtered and stored at −80 °C for HPLC analysis. Where indicated, media was supplemented with amino acids valine, isoleucine or threonine (~20 mM).

*Bioreactor fermentation* Recombinant *E. coli* strains were streaked on LB agar plates plus antibiotic (Ampicillin 100 µg/mL) and incubated for 16 h at 37 °C. A single colony was picked and used to inoculate a 250 mL baffled flask containing 50 mL of minimal medium plus antibiotics. The culture was grown overnight in a shaking incubator at 37 °C and 200 rpm and used to inoculate flasks containing 250 mL of minimal medium plus antibiotics and arabinose (10 mM). The volume of inoculum was adjusted depending on the desired initial OD_600_. All fermentations were performed in duplicate in 400 mL stirred tank DasGip reactors (250 mL operation volume). Temperature and pH were maintained at 30 °C and 7.0, respectively. pH was controlled by the addition of a 4 N NaOH solution. Prior to inoculation, the medium was flushed with N_2_ for 20 min. After inoculation, N_2_ was top-sparged at a constant rate. For aerobic conditions, dissolved oxygen (DO) was maintained at 80% of saturation using a cascade control linking airflow and agitation speed. Initial flow was set at 10 L/h. Samples were regularly taken, and optical OD_600_ was monitored every 3 h. Samples were filtered and stored at −80 °C for HPLC analysis.

### HPLC analysis

Organic acids, carbohydrates, and alcohol were quantified by ion-exclusion chromatography using an Agilent 1200 HPLC system and an Agilent Hi-Plex H column (300 × 7.7 mm, PL1170-6830) with a guard column (SecurityGuard Carbo-H, Phenomenex PN: AJO-4490). Sugars and alcohols were monitored using a refractive index detector (Agilent RID, G1362A), set on positive polarity and optical unit temperature of 40 °C. Organic acids were monitored either with RI and/or UV at 210 nm (Agilent MWD, G1365B). 30 µL of the sample was injected onto the column using an autosampler (Agilent HiP-ALS, G1367B), and column temperature kept at 40 °C using a thermostatted column compartment (Agilent TCC, G1316A). Analytes were eluted isocratically with 14 mM H_2_SO_4_ at 0.4 mL/min for 50 min. Chromatograms were integrated using ChemStation software (REV: B.03.02). A previously reported method [18] was also used for comparison.

### Protein extraction, trypsin digestion, and identification

Samples (2 mL) were collected from the reactor before anaerobic induction and 1 h after induction. Samples were centrifuged at 4 °C, 17,000×*g* for 20 min. The pellet was resuspended in 300 µL of BugBuster MasterMix solution (Novagen), and intracellular proteins were recovered according to the supplier’s instructions. Protein quantification was performed with a 2D-Quant kit (GE Healthcare). Protein extracts were stored at −80 °C before trypsin digestion. Trypsin digestion was performed as described previously [30]. Reverse-phase chromatography was used to clean the digested peptide mixtures using C-18 Zip Tip eluting with 70% acetonitrile (v/v). Residual acetonitrile was removed by vacuum centrifugation (Eppendorf, Hamburg, Germany) and peptides resuspended in 0.1% formic acid before analysis. Peptide identification was performed using LC–MS/MS (AB Sciex 5600, Ontario, Canada). The LC system was equipped with a Vydac MS C18 300-Å, 150 mm × 0.3 mm column (Grace Davison Discovery Sciences, Deerfield, IL) operated at 30 °C with a 0–80% acetonitrile gradient (in 0.1% formic acid) for 105 min at a flow rate of 3 μL/min as described in [31]. Protein Pilot 4.2 software (Applied Biosystems, Foster City, CA) was used to identify all proteins. The mass tolerance values for precursor ions and fragment ions were set to the default values of the Paragon search algorithm and trypsin was specified as the digesting protease. The complete genome sequence for *E. coli* K-12 MG1655 was used as sequence database with the addition of the sequence of the optimised gene for the methylmalonyl-CoA epimerase from *P. acidipropionici*. Hits were considered positive when at least two peptides with more than six residues and 90% confidence were detected.

## Results

### *In silico* analysis

*The genome*-*scale model, i*JO1366, *predicts that PA can be natively produced* The *E. coli* GEM, iJO1366 [26] was used to test the native metabolism of *E. coli* for PA biosynthesis. The model predicts glucose is catabolised to acetate, ethanol, and formate in a molar ratio of 1:1:2.1 during anaerobic growth (Table 3). Traces of succinate (about 1% of the total acetate produced on a mole basis) were also produced as a result of lysine and methionine biosynthesis. The original model only permits sodium-driven import of PA. Since PA is permeable to the *E. coli* membrane [32], we added an export mechanism. The addition of the transporter enables PA production. Based on the pKa of PA, the transporter chosen for modelling was a proton symport. The model predicts conversion of succinate to PA through the *sbm* operon. PA production is energetically favourable given that succinate transport utilises a proton antiport mechanism. Additional simulations using the parsimonious assumption predicted a significant redistribution of fluxes through the central carbon metabolism: acetate, ethanol, formate and propionate produced at a ratio of 1:0.8:1.9:0.2 (S1, Table 3). Furthermore, the solution space shows that when the objective function is set to maximise for maintenance, 2.5 ATP/glucose can be yielded from producing acetate, ethanol, and formate in a 1:1:2 ratio or acetate, formate, and propionate in a 1:1:1 ratio. This simulation suggests that *E. coli* may produce PA without energetic penalty using a functional *sbm* operon.

**Table 3:** Overall Estimated Yields (mmol)

	Wild type^a^	S1^b^
Biomass	0.2323	0.2331
Acetate	8.2337	8.2661
CO_2_	0	0
Ethanol	8.1936	6.1550
Formate	17.3023	15.3765
Glucose	−10	−10
O_2_	0	0
Propionate	0	2.0708
Succinate	0.0770	0

^a^ Original model including energetic parameter changes (removal of the polyphosphate kinase reaction), maximised for growth under the parsimonious FBA assumption

^b^ Addition of the propionate symport transporter maximised for growth rate under the parsimonious FBA assumption showing predicted propionate production using the *sbm* operon

*The maximum theoretical yields of PA is 0.85 C*-*mol/C*-*mol glucose* The model was used to calculate the maximum theoretical yields for PA with and without the *sbm* reactions. When the reactions encoded in the *sbm* operon are present in the model, *E. coli* utilises a combination of the pentose phosphate and the Entner-Doudoroff pathways as well as the TCA cycle with glyoxylate bypass to source additional reduced cofactors for PA production. Succinate is produced through the phosphoenolpyruvate carboxylase and the dicarboxylic branch of the TCA cycle, allowing for a net PA production of ~0.85 C-mol/C-mol glucose. The ratio drops to ~0.33 C-mol/C-mol glucose (0.67 mol PA per mole glucose) when the cycle is inactive, forcing PA production through the energy intensive synthesis and subsequent degradation of threonine.

### Heterologous PA production

*The native sbm operon cannot produce PA in minimal medium* To determine if the expression of the *sbm* operon was enough for the production of PA in vivo, we reproduced results from [20, 21] using a plasmid expression system controlled by the T7 promoter (pET28a_sbm) in complex media. PA production was measured after fermenting cells in serum bottles using the same conditions described previously [19–21]. The authors suggest that a high starting OD_600_ results in high PA titers and they showed that above 10 OD_600_ units, changes in the PA/1-propanol titers were not significant. Thus, 10 OD_600_ units were used. While PA was detected, the levels were far lower than the previously reported and substantial production of lactate was observed. Table 2 shows the final fermentation products for the batch fermentation in serum bottles. Biomass production was higher in complex media (0.54 g/L) compared to minimal media (0.33 g/L); the major fermentation product was lactate in both conditions (~30 mM). PA was only detected in complex media (0.34 ± 0.02 mM). Ethanol production was similar in both conditions (~11 mM), however the acetate to ethanol ratio was nearly 1:2, compared to the expected 1:1 ratio. That could be associated to a stress response.

To validate our results, a sample at the highest PA concentration was compared using two HPLC methods (Additional file 1: Figure S1). The first method, described in [19], runs for 20 min and displayed a ghost peak co-eluting with PA (Additional file 1: Figure S4). To resolve the peaks, the second method runs for 50 min with retention times for PA and 1-propanol at ~28.1 min and ~41.4 min, respectively. This method resolved the ghost peak co-eluting with PA, and careful evaluation of the HPLC data showed that only one of the two peaks aligned with the PA standard. The total amount of PA estimated was considerably lower when using the long method (Table 4). Notably, the ghost peak was only present when complex medium was used. No changes in the final concentration of organic acids were observed even after 80 h and lactate was produced. 1-Propanol was detected below the limit of quantitation level.

**Table 4:** Fermentation products for *E. coli* BL21 (DE3) harbouring plasmid *pPET28*_*sbm*

	Glucose^a^	Pyruvate	Succinate	Lactate	Formate	Acetate	Propionate	Ethanol	Biomass^b^
Mineral medium	22.93 ± 2.75	1.10 ± 0.07	1.88 ± 0.09	30.90 ± 3.39	11.66 ± 0.89	5.78 ± 0.32	ND	11.10 ± 0.28	0.33 ± .04
Complex medium	26.49 ± 2.54	0.64 ± 0.04	1.17 ± 0.10	33.59 ± 4.03	15.31 ± 0.68	7.47 ± 0.73	0.34 ± 0.02	11.56 ± 0.64	0.54 ± .08

Data correspond to the average of two replicates. All concentration are reported in mM. ND: not detected or below the limit of detection

^a^ Data reported correspond to total glucose consumed out of 100 mM

^b^ Biomass is reported in g/L

*Co*-*expression of the sbm operon with an epimerase leads to PA production in minimal medium* PA and 1-propanol were only produced in complex medium (Table 4) suggesting that PA precursors are derived from amino acids catabolism rather than glucose. The model assumes that ScpA converts succinyl-CoA into (S)-methylmalonyl-CoA, the substrate for ScpB (Additional file 2), whereas experimental data suggests ScpA converts succinyl-CoA into (R)-methylmalonyl-CoA [24, 25] (Fig. 1). To investigate if the native succinate dissimilation pathway is restricted by the conversion of the (R)-methylmalonyl-CoA to the (S)-enantiomer, the methylmalonyl-CoA epimerase gene (*mce*) from *P. acidipropionici* was co-expressed (pET28_sbm-mce).

A strain harbouring both the *sbm* operon and the epimerase was grown on minimal medium (Table 5). Growth was observed for 24 h, and glucose consumption continued for 48 h at which point pH had dropped to 5.22 ± 0.28. In all four replicates, PA was produced at a concentration of 0.23 mM. 1-Propanol was detected below the limit of quantification of the HPLC method.

**Table 5:** Fermentation products for *E. coli* BL21 (DE3) harbouring plasmid *pPET28*_*sbm*-*mce*

	Glucose^a^	Pyruvate	Succinate	Lactate	Formate	Acetate	Propionate	Ethanol	Biomass^b^
Mineral medium	72.01 ± 3.96	0.76 ± 0.04	8.11 ± 0.37	65.03 ± 2.99	24.15 ± 1.11	15.16 ± 0.69	0.23 ± 0.02	19.37 ± .92	0.85 ± 0.04

Data correspond to the average of four replicates. All concentration are reported in mM

^a^ Data reported correspond to total glucose consumed out of 100 mM

^b^ Biomass is reported in g/L

**Fig. 2:**
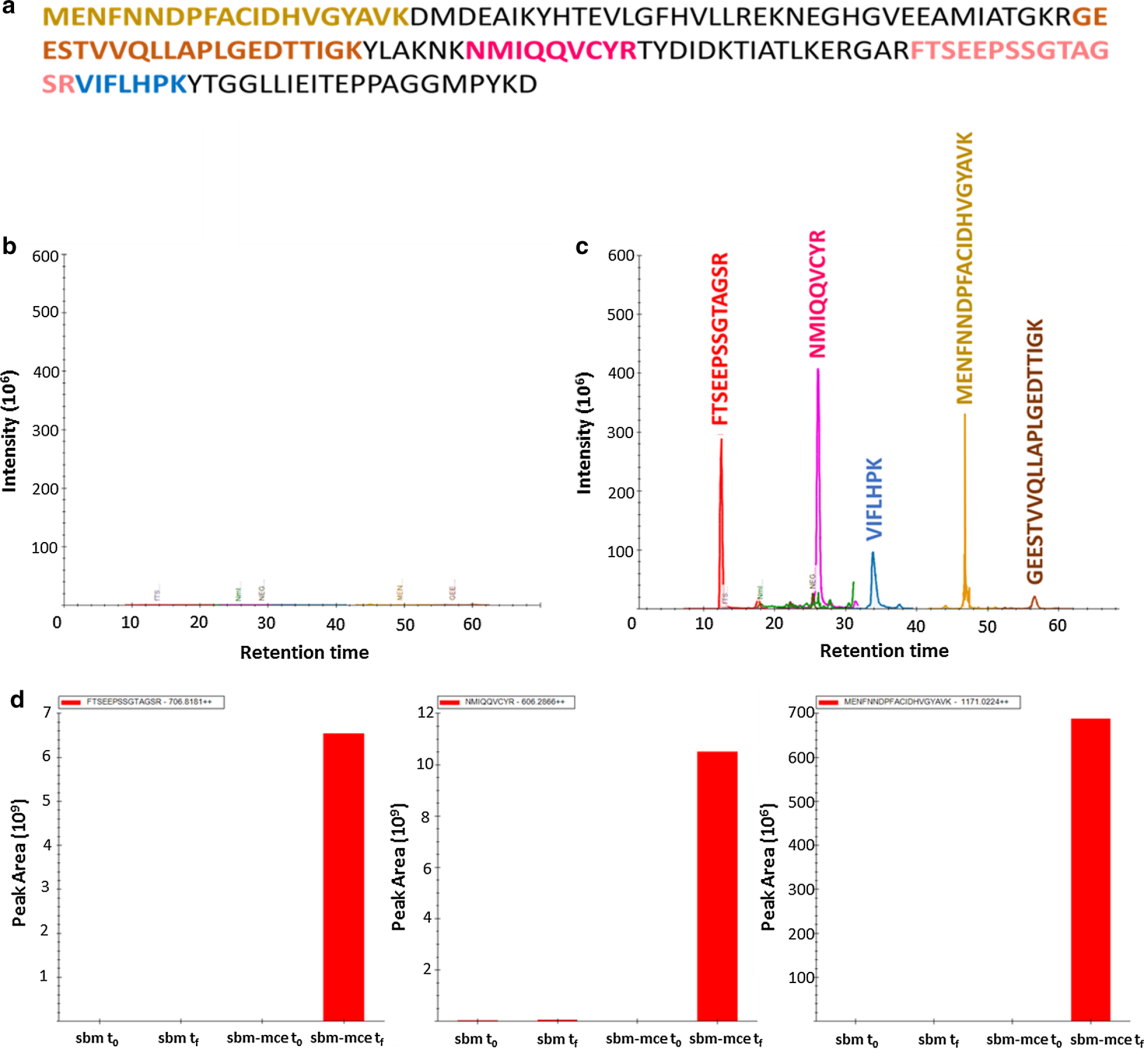
Protein discovery for the methylmalonyl CoA epimerase. **a** Protein sequence. The most abundant peptides have been highlighted. **b** Chromatograms corresponding to peptide identification for cultures harbouring plasmid pBRP_BAD__sbm. **c** Chromatograms corresponding to peptide identification for cultures harbouring plasmid pBRP_BAD__sbm-mce. The three most abundant peaks to peptides FTSEEPSSGTAGSR (*red*), NMIQQVCYR (*pink*), and MENFNNDPFACIDHVGYAVK (*yellow*). **d** Peak areas comparison for the three most abundant peptides in samples taken from cultures at the beginning and the end of the fermentations

To verify that the simultaneous expression of the epimerase and the *sbm* operon genes lead to PA production in minimal media, *E. coli* strain DH5-α was transformed with a plasmid containing the sbm operon (pBRP_BAD__sbm) and a plasmid containing both the *sbm* operon and *mce* gene (pBR_BAD__sbm-mce). Batch cultures were induced from the beginning with arabinose (10 mM) and proteins were extracted from samples taken at the beginning and the end of the fermentation as described elsewhere [33]. Proteins were identified using nano-LC–MS after trypsin digestion (Fig. 2). PA was only detected in cultures harbouring plasmid pBRP_BAD__sbm-mce (0.5 mM). Peptides corresponding to all enzymes of the *sbm* operon were identified in both cultures (data not shown) and seven peptides from Mce were identified in cultures harbouring plasmid pBRP_BAD__sbm-mce (Fig. 2c). The comparative expression of pBRP_BAD__sbm and pBRP_BAD__sbm-mce, as shown by the presence of the Mce peptides after induction illustrates that the expression of *mce* is required for propionate production from the *sbm* operon.

**Fig. 3:**
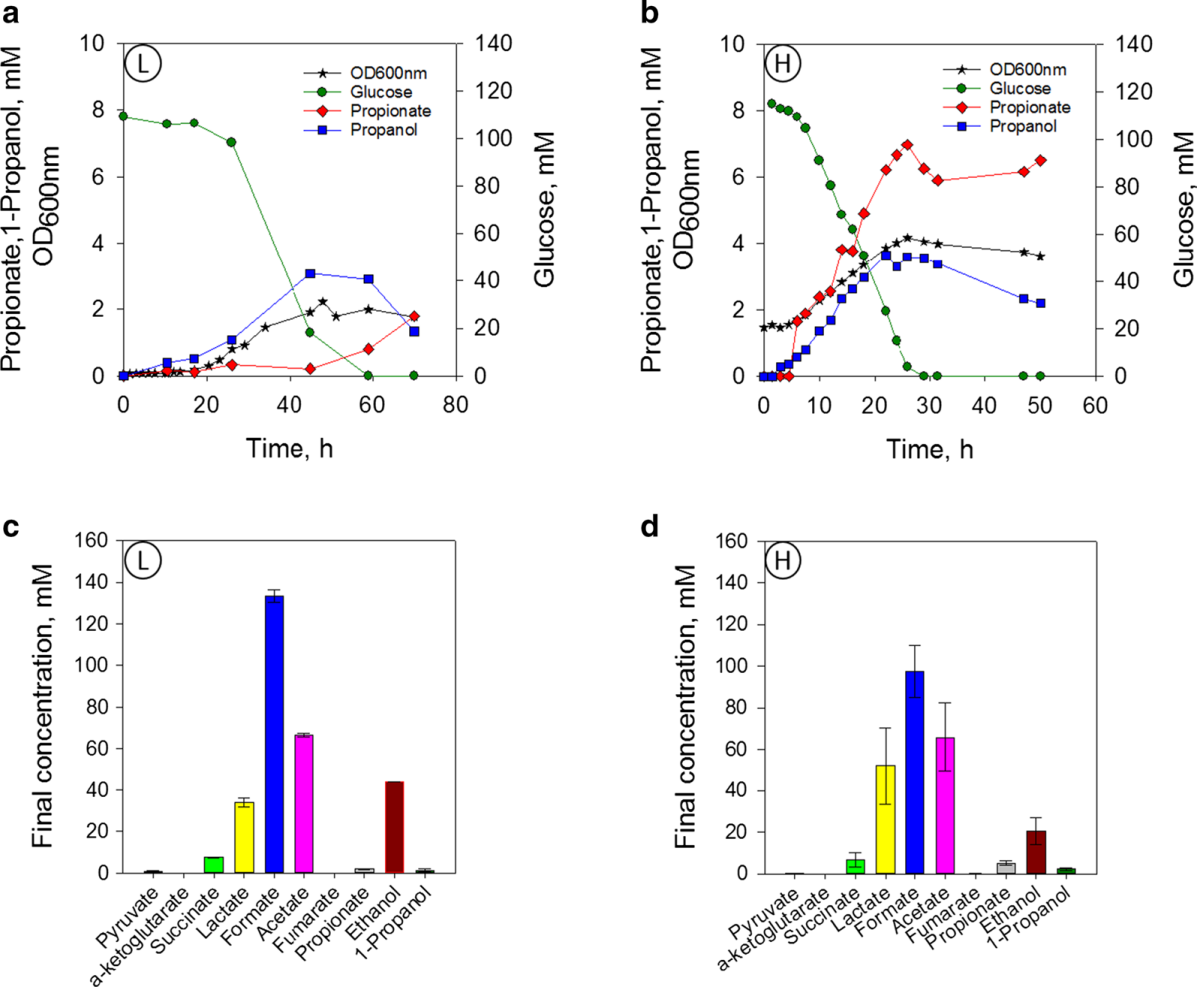
PA production in 250 mL DASGIP bioreactor under anaerobic conditions. Arabinose was added to the media from the beginning of the fermentation. Results correspond to the average of two replicates. **a** Fermentation profile for culture starting at low OD = 0.10 (L). **b** Fermentation profile for culture starting at high OD = 1.00 (H). **c** Final organic acids concentration for fermentation starting at low OD = 0.10 (L). **d** Final organic acids concentration for fermentation starting at high OD = 1.0 (H)

*The use of a medium strength promoter (P*_*BAD*_ *) improved PA production* The T7 promoter was replaced by a medium strength promoter, P_BAD_, to test the hypothesis that the metabolic burden caused by the T7 promoter and the adverse effects of IPTG limited cell growth and reduced PA production. The plasmid pBRP_*BAD*__sbm-mce was used to transform the DH5-α strain. Two starting biomass concentrations were tested: low OD_600_ (≈0.1) and high OD_600_ (≈1.0) (Fig. 3). Low starting biomass cultures displayed two times higher growth rates compared to high starting biomass cultures (0.139/h vs. 0.059/h). However, more biomass was produced in the cultures that started at higher initial biomass (2.16 g/L vs. 1.67 g/L). The final PA titer was three times higher in cultures starting at higher biomass, reaching a final concentration of 6.5 mM compared to 1.79 mM. For the high biomass condition, the yield was 22.9 mg_PA_/g_glucose_, and the productivity was 12.0 mg/L/h compared to 13.7 mg_PA_/g_glucose_ and a productivity of 6.1 mg/L/h in the low biomass condition.

1-Propanol and PA were detected from the beginning of the fermentation. In both conditions, 1-propanol was produced steadily during the fermentation, while PA production was delayed at the culture that started at low biomass. In both conditions, the highest 1-propanol concentration (3.5 mM) was reached just before glucose depletion. After this stage, 1-propanol concentration decreased which correspond to a small increase in PA. PA concentration increased in a 2-fold after glucose was depleted at the low biomass conditions. In the cultures that started at high biomass, PA concentration remained around 6 mM after glucose depletion. Formate, acetate, and ethanol were the primary fermentation products with traces of lactate and succinate also detected.

**Fig. 4:**
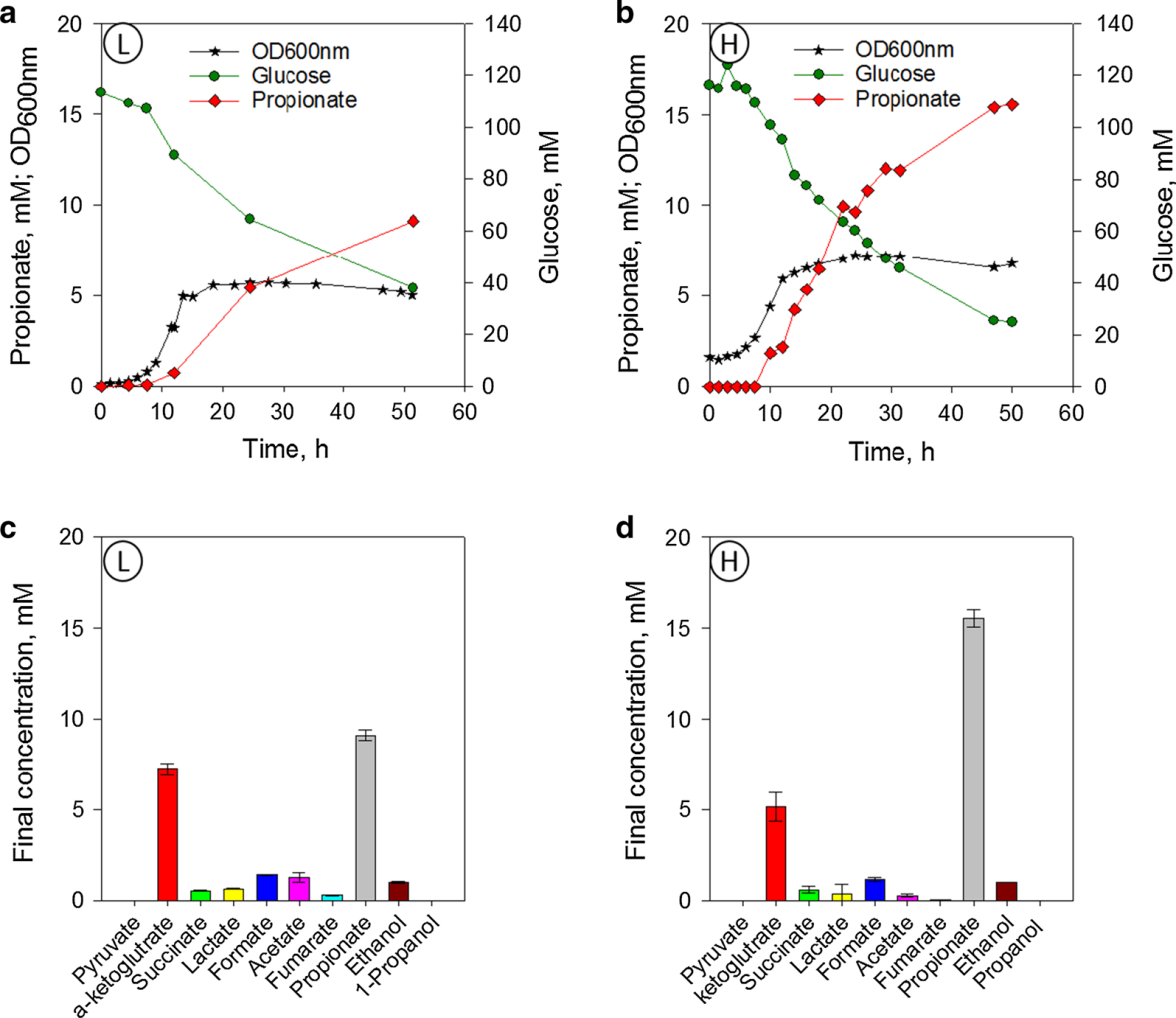
PA production in 250 mL DASGIP bioreactor under aerobic conditions. Arabinose was added to the media from the beginning of the fermentation. Results correspond to the average of two replicates. **a** Fermentation profile for culture starting at low OD = 0.10 (L). **b** Fermentation profile for culture starting at high OD = 1.00 (H). **c** Final organic acids concentration for fermentation starting at low OD = 0.10 (L). **d** Final organic acids concentration for fermentation starting at high OD = 1.0 (H)

*Aerobic cultures enhance PA yield E. coli* DH5-α harbouring pBRP_*BAD*__sbm-mce was grown under aerobic conditions, which would provide a higher available pool of succinyl-CoA as consequence of a highly active TCA cycle [34]. Again two starting biomass concentrations were evaluated (Fig. 4). PA production started after 10 h and was 1.5 times higher compared to anaerobic conditions. For the low starting OD cultures, cells reached the stationary phase after 20 h. The fermentation was monitored for a total of 50 h (after 50 h, no changes in biomass concentration were observed). At this point 64% of total glucose supplied was consumed. Final PA concentration was 9.12 mM. The second major fermentation product was α-ketoglutarate. Low levels of succinate, lactate, acetate, fumarate and ethanol were produced at less than 2 mM. 1-Propanol was not detected in the media. Similarly to the anaerobic conditions, a high initial OD favoured PA production in aerobic conditions. Glucose consumption, PA, and biomass production were higher than the low starting biomass culture (Fig. 4). Stationary phase was reached at 15 h. Compared to the best results reached in anaerobic conditions, aerobic fermentation increased PA yield by 3-fold from 22.9 mg_PA_/_glucose_ to 70.5 mg_PA_/_glucose_. Productivity was also increased nearly 5-fold from 6.1 to 28.7 mg/L/h.

*The sbm operon promotes production of PA from amino acid degradation* Based on our experimental observations, PA was not produced from glucose when the epimerase is not co-expressed with the operon. We speculated that in complex media, PA was produced from the degradation of amino acids. Complex media contains peptides and amino acids. It is well known that *E. coli* can degrade threonine to PA [35]. In *S. erythraea,* a genome-scale analysis showed that three amino acids (threonine, isoleucine, and valine) could supply propionyl-CoA and (R/S)-methylmalonyl-CoA to enhance erythromycin production [36]. In addition, enzymes that belong to the crotonase superfamily are expected to perform a wide range of reactions [37]. Thus, we tested the potential of the individual expression of the *sbm* operon to use the aforementioned amino acids.

**Fig. 5:**
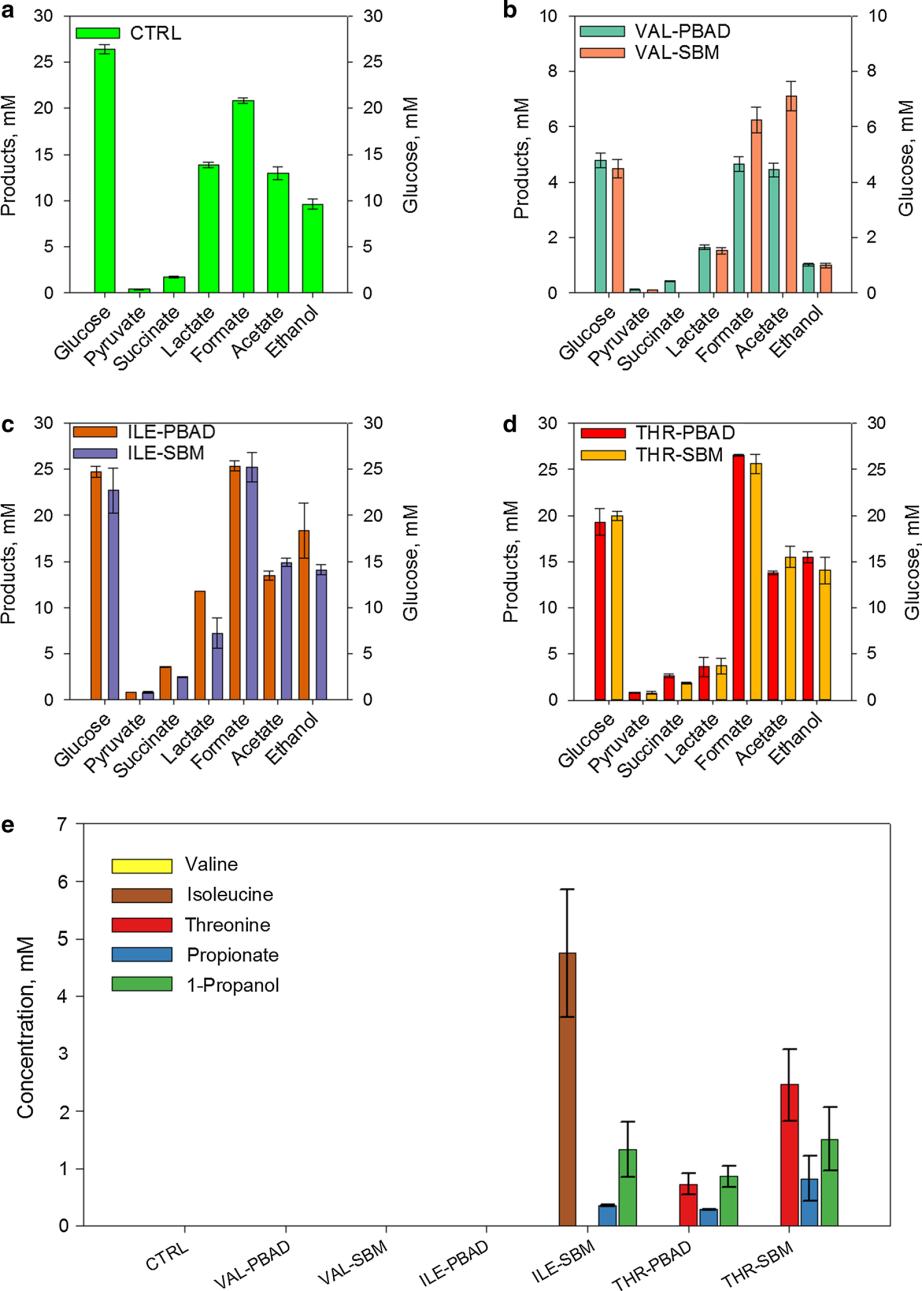
PA production from amino acids degradation in anaerobic fermentation. Experiments were performed in serum bottles with strains harbouring empty plasmid (PBAD) and the *sbm* operon genes (SBM). **a** Total glucose consumption and final organic acids profile for control strain (CTRL, no plasmid). **b** Total glucose consumption and final organic acid profile for cultures supplemented with valine (VAL). **c** Total glucose consumption and final organic acids profile for cultures supplemented with isoleucine (ILE). **d** Total glucose consumption and final organic acids profile for cultures supplemented with threonine (THR). **e** Total amino acids consumption and final PA and 1-propanol concentration. Data correspond to the average of two replicates

*Escherichia coli* DH5-α, harbouring no plasmid (CTRL), the empty plasmid pPBRP_BAD_ (PBAD) and plasmid pBRP_BAD__sbm (SBM) were grown under anaerobic condition in serum bottles in minimal medium. Three media supplemented with valine, isoleucine or threonine were prepared at an initial concentration of ~20 mM. The growth profiles for all tested conditions was conserved (Additional file 3). CTRL culture showed the typical mixed-acid fermentation for *E. coli.* This culture did not show production of PA or 1-propanol, in agreement with all reports regarding the inability of *E. coli’s* native metabolism to produce PA. Valine supplemented cultures (VAL) consumed less than 6 mM of glucose (Fig. 5) and growth was inhibited as previously reported [38]. The average glucose consumption for the other cultures was ~23 mM. After 40 h, propionate was detected in the cultures supplemented with and threonine (THR) but also in the cultures supplemented with isoleucine (ILE) where the *sbm* operon was expressed. The culture ILE-SBM consumed 4.7 mM of isoleucine, and produced 0.36 mM of PA and 1.34 mM of 1-propanol. As previously reported [35], there was PA production by native *E. coli* metabolism from threonine degradation (THR-PBAD) as seen if Fig. 5e (0.29 mM PA, 0.86 mM 1-propanol). However the expression of the *sbm* operon increased PA and 1-propanol production by 2-fold up to 0.82 and 1.51 mM, respectively. Threonine consumption was increased from 0.73 to 2.45 mM (Fig. 5).

## Discussion

Haller et al. [23] suggested that the *sbm* operon provides an alternative succinate dissimilation pathway to PA. This would require that *E. coli* mutase (ScpA), unlike other known mutases [24], would produce the (S)-enantiomer of methylmalonyl-CoA directly or that the methylmalonyl-CoA decarboxylase acts on the (R)-enantiomer (Fig. 1). The authors were unable to directly validate this claim as their HPLC method was unable to distinguish between the (R) and (S)-enantiomer of methylmalonyl-CoA. However, they did provide indirect evidence through a coupled assay of ScpA and ScpB that it was operational. Other studies have concluded that ScpA is similar to most mutases and can only catalyse the conversion between succinyl-CoA and the (R)-methylmalonyl-CoA enantiomer. Dayem et al. [25] confirmed initial results from Vallari et al. [39] that *E. coli* does not produce the (S)-methylmalonyl CoA enantiomer [40]. More recently, Li et al. [22] engineered a succinate producing strain T110 and expressed the *sbm* operon and the *ABb* operon from *Methylobacterium extorquens* AM1 to produce PA. Although a different HPLC method was used, the results from Li et al. [22] show titres in the same order of magnitude for PA production when the *sbm* operon is expressed in complex media (Table 2). Further engineered on strain T110 showed a 3-fold increase in PA titre when the *sbm* operon was expressed. The authors reported increased titres up to 0.49 mol PA/mol glucose when operon *ABb* was co-expressed. Those results show that the three additional enzyme could perform the epimerase step required to complete the production of PA. Simulations showed that if the native *sbm* operon was functional, *E. coli* could ferment sugars into acetate, formate and PA instead of the typical acetate, ethanol, and formate byproduct commonly obtained, as both profiles are energetically equivalent. Nonetheless, when we expressed the *sbm* operon and cultured cells in minimal media, PA was not produced unless the epimerase was co-expressed. This result supports previous observations [24, 39], and shows that the mutase only produces the (R)-enantiomer. Our results cast doubts upon previous works on the expression of the *sbm* operon for PA production [18–21]. In addition to the epimerase required to complete the succinate dissimilation towards PA, the organic acid could still be produced if a source of (S)-methylmalonyl-CoA or propionyl-CoA was available (e.g. from the degradation of amino acids or peptides) [35]. While threonine degradation in *E. coli* is known to produce PA, the expression of the *sbm*-operon doubled PA production. *E. coli* is natively unable to degrade isoleucine as shown by the *E. coli* PBAD-ILE culture. However, when the *sbm* operon is active (SBM-ILE), isoleucine is metabolised. We attribute this feature to the promiscuous activity of the crotonase (ScpB). Our results show that the presence of an unresolved ghost peak as well as the use of complex media have contributed to the PA levels previously reported by other authors [18–22].

After culturing the strain harbouring both the *sbm* and the *mce* in minimal medium, the final PA concentration was close to the concentration obtained in complex media. However, the yield observed was lower than the model predictions (Table 3). This was attributed to high lactate production. Lactate is produced as a stress response from the strong T7 promoter [40] as evidenced by the biproduct profile which comprised succinate, ethanol, and lactate. It is well known that the use of a strong T7 promoter is detrimental for metabolic engineering [41, 42]. The use of the arabinose inducible promoter *P*_*BAD*_ offered a double advantage for PA production: on the one hand, it allowed a moderate regulation of gene expression, and at the same time it helped with the catabolic repression of glucose. By enabling biomass accumulation before the production of a toxic metabolite, a 30-fold increase in production was achieved under anaerobic conditions.

PA production is tightly linked to succinate biosynthesis. It is well accepted that anaerobic *E. coli* fermentation products depend on the balance of reducing power, which results in ethanol, formate, acetate and lactate production as major products [43], leading to a reduction of the succinate and the succinyl-CoA pools. In the case of DH5α strain, which native metabolism has not been engineered to provide an optimal performance for succinate production under anaerobic conditions, aerobic conditions provide a steady pool of succinate and succinyl-CoA through a high TCA-flux and the regeneration of cofactors. Here, the shift from anaerobic to aerobic conditions led to a 67-fold increase in PA production from the initial T7 promoter-based system (0.23 to 15.53 mM). Despite such improvement, the production remained modest compared to native producers [44] and the engineered *E. coli* strain T110 [22]. Production of 1-propanol reached a final concentration of ~4 mM indicating a high promiscuous activity of the AdhE on propionyl-CoA [45].

Under anaerobic conditions 1-propanol seems to be reassimilated after reaching a peak which coincides with glucose depletion and a slight increase in PA production. This is likely the result of a detoxification mechanism [46] induced by 1-propanol. Both low and high biomass conditions showed similar profiles for 1-propanol production, which suggests that cells are able to sense a certain concentration of 1-propanol which triggers a mechanism for detoxification. Our data also shows that the accumulation of organic acids and ethanol play an important role in activating mechanisms of acid and alcohol tolerance [47].

## Conclusion

The presence of a silent operon in *E. coli* for the production of PA has long intrigued scientists. This pathway, however, is not energetically beneficial compared to lactate and ethanol, which provide a faster and cheaper way to balance redox. Still, PA production can be improved by further engineering of the native metabolism forcing cells to direct carbon towards the production of succinate as described and performed recently [22]. However, the small energetic benefit from avoiding the succinate antiporter is rapidly offset by the requirements to synthesise enzymes for a long catabolic pathway. It is likely that this disadvantage is one of the reasons for which, evolution has silenced the operon. Under aerobic conditions, higher PA production is possible because the TCA cycle enables redox balancing, thus not limiting redox or energy. The toxic nature of PA may be another possible explanation for the pathway inactivation. The inhibitory nature of the propionyl-CoA and succinyl-CoA may play a similar role.

## Additional files

**Additional file 1: Figure S1.** Chromatogram for the HPLC standards ran in the method reported in [20]. UV detectors signal (A) and RI detector signal (B) are showed. Red line indicates the standards, and blue line indicates the default baseline. Propionate is detected at 17.77 min and 18.066 min in the UV and RI detector, respectively.** Figure S2.** Chromatogram for the HPLC standards ran in the method reported in this work. UV detectors signal (A) and RI detector signal (B) are showed. Red line indicates the standards and blue line indicates the default baseline. Propionate is detected at 28.01 min and 28.396 min in the UV and RI detector, respectively.** Figure S3.** Chromatogram for sample taken from an anaerobic culture of E. coli BL21 harbouring plasmid pET28a+_sbm after 48 h. The HPLC method used was the reported in [20]. UV detectors signal (A) and RI detector signal (B) are showed. Blue line indicates the default baseline. Red and green lines are replicates for the same sample. UV detector showed propionate, but this peak does not appear in RI. Instead, there was a signal at 17.18 min.** Figure S4.** Chromatogram for sample taken from an anaerobic culture of E. coli BL21 harbouring plasmid pET28a+_sbm after 48 h. The HPLC method used was the reported in this work. UV detectors signal (A) and RI detector signal (B) are showed. Blue line indicates the default baseline. Red and green lines are replicated for the sample. UV signal shows that the propionate peak eluting in the short method correspond to two peaks. No propionate was present in the sample. In the RI detector, propionate (28.404 min) elutes closely to another peak (28.42 min). This peak later increases (data not shown in the chromatogram).

**Additional file 2: Table TS1.** Base model with epimerase.** Table TS2.** Base model without epimerase. Table TS3. Flux distribution.

**Additional file 3: Table SA-A.** Control Fermentation.** Table S3-B.** Valine.** Table S3-C.** Isoleucine. ** Table S3-D.** Threonine.

## Abbreviations

GLC: glucose
DHAP: dihydroxyacetone phosphate
PEP: phosphoenolpyruvate
PYR: pyruvate
ETOH: ethanol
ACCOA: acetyl-CoA
ACE: acetate
OAA: oxaloacetate
ISOC: isocitrate
A_KETO: α-ketoglutarate
SUCCOA: succinyl-CoA
SUC: succinate
FUM: fumarate
MAL: malate
MMCOA-S/R: (R/S)-methylmalonyl-CoA
PCOA: propionyl-CoA
PROP: propionate
PA: propionic acid
OD: optical density
TCA: tricarboxylic acids cycle
