# Supplementary material for: Awakening sleeping beauty: production of propionic acid in Escherichia coli through the sbm operon requires the activity of a methylmalonyl-CoA epimerase
Source: Microb Cell Fact. 2017 Jul 17;16:121. doi: 10.1186/s12934-017-0735-4 (PMC5512728; doi:10.1186/s12934-017-0735-4)
Supplement: Supplementary file 1 — Additional file 1: Figure S1. Chromatogram for the HPLC standards ran in the method reported in [20]. UV detectors signal (A) and RI detector signal (B) are showed. Red line indicates the standards, and blue line indicates the default baseline. Propionate is detected at 17.77 min and 18.066 min in the UV and RI detector, respectively. Figure S2. Chromatogram for the HPLC standards ran in the method reported in this work. UV detectors signal (A) and RI detector signal (B) are showed. Red line indicates the standards and blue line indicates the default baseline. Propionate is detected at 28.01 min and 28.396 min in the UV and RI detector, respectively. Figure S3. Chromatogram for sample taken from an anaerobic culture of E. coli BL21 harbouring plasmid pET28a+_sbm after 48 h. The HPLC method used was the reported in [20]. UV detectors signal (A) and RI detector signal (B) are showed. Blue line indicates the default baseline. Red and green lines are replicates for the same sample. UV detector showed propionate, but this peak does not appear in RI. Instead, there was a signal at 17.18 min. Figure S4. Chromatogram for sample taken from an anaerobic culture of E. coli BL21 harbouring plasmid pET28a+_sbm after 48 h. The HPLC method used was the reported in this work. UV detectors signal (A) and RI detector signal (B) are showed. Blue line indicates the default baseline. Red and green lines are replicated for the sample. UV signal shows that the propionate peak eluting in the short method correspond to two peaks. No propionate was present in the sample. In the RI detector, propionate (28.404 min) elutes closely to another peak (28.42 min). This peak later increases (data not shown in the chromatogram). [file 12934_2017_735_MOESM1_ESM.docx]

A

B

**Figure S1.** Chromatogram for the HPLC standards ran in the method reported in [**20**]. UV detectors signal (A) and RI detector signal (B) are showed. Red line correspond to the standards and blue line correspond to the default baseline. Propionate is detected at 17.77 min and 18.066 min in the UV and RI detector, respectively.

A

B

**Figure S2.** Chromatogram for the HPLC standards ran in the method reported in this work. UV detectors signal (A) and RI detector signal (B) are showed. Red line correspond to the standards and blue line correspond to the default baseline. Propionate is detected at 28.01 min and 28.396 min in the UV and RI detector, respectively.

A

B

**Figure S3.** Chromatogram for a sample taken from an anaerobic culture of *E. coli* BL21 harbouring plasmid pET28a+_sbm after 48 h. The HPLC method used was the reported in [**20**]. UV detectors signal (A) and RI detector signal (B) are showed. Blue line correspond to the default baseline. Red and green lines are replicates for the sample. UV detector showed propionate, but this peak does not appear in RI. Instead, there was a signal at 17.18 min.

A

B

**Figure S4.** Chromatogram for a sample taken from an anaerobic culture of *E. coli* BL21 harbouring plasmid pET28a+_sbm after 48 h. The HPLC method used was the reported in this work. UV detectors signal (A) and RI detector signal (B) are showed. Blue line correspond to the default baseline. Red and green lines are replicated for the sample. UV detector showed that the propionate peak eluting in the short method corresponds actually to two peaks. No propionate was present in the sample. In the RI detector, propionate (28.404 min) elutes closely to another peak (28.42 min). This peak later increases (data not shown in the chromatogram)
